# Perioperative and anesthetic risk factors of surgical site infection in patients undergoing pancreaticoduodenectomy: A retrospective cohort study

**DOI:** 10.1371/journal.pone.0240490

**Published:** 2020-10-14

**Authors:** Saori Yamamoto, Yusuke Nagamine, Tetsuya Miyashita, Shiono Ito, Yurika Iwasawa, Michihiko Kawai, Shinsaku Saito, Tomohisa Tamai, Takahisa Goto

**Affiliations:** Department of Anesthesiology and Critical Care Medicine, Yokohama City University Hospital, Yokohama, Kanagawa, Japan; China Medical University Hospital, TAIWAN

## Abstract

**Background:**

Surgical site infection is a major perioperative issue. The morbidity of surgical site infection is high in major digestive surgery, such as pancreaticoduodenectomy. The comprehensive risk factors, including anesthetic factors, for surgical site infection in pancreaticoduodenectomy are unknown. The aim of this study was to investigate the perioperative and anesthetic risk factors of surgical site infection in pancreaticoduodenectomy.

**Methods:**

This was a retrospective cohort study conducted in a single tertiary care center. A total of 326 consecutive patients who underwent pancreaticoduodenectomy between January 2009 and March 2018 were evaluated. Patients who underwent resection of other organs were excluded. The primary outcome was the incidence of surgical site infection, based on a Clavien-Dindo classification of grade 2 or higher. Multivariable logistic regression analysis was performed to investigate the association between surgical site infection and perioperative and anesthetic factors.

**Results:**

Of the 326 patients, 116 (35.6%) were women. The median age was 70 years (interquartile range; 64–75). The median duration of surgery was 10.9 hours (interquartile range; 9.5–12.4). Surgical site infection occurred in 60 patients (18.4%). The multivariable analysis revealed that the use of desflurane as a maintenance anesthetic was associated with a significantly lower risk of surgical site infection than sevoflurane (odds ratio, 0.503; 95% confidence interval [CI], 0.260–0.973). In contrast, the duration of surgery (odds ratio, 1.162; 95% CI, 1.017–1.328), cerebrovascular disease (odds ratio, 3.544; 95% CI, 1.326–9.469), and ischemic heart disease (odds ratio, 10.839; 95% CI, 1.887–62.249) were identified as significant risk factors of surgical site infection.

**Conclusions:**

Desflurane may be better than sevoflurane in preventing surgical site infection in pancreaticoduodenectomy. Cerebrovascular disease and ischemic heart disease are potential newly-identified risk factors of surgical site infection in pancreaticoduodenectomy.

## Introduction

Surgical site infection refers to infection of the skin and subcutaneous tissue at the incision, organ, or space that occurs after surgery [1]. Surgical site infections increase the length of hospital stay, reoperation rate, and number of in-hospital deaths, and thus greatly affects the postoperative outcome of patients undergoing surgery [2, 3]. Furthermore, it increases medical costs [2, 4, 5]. Gastrointestinal surgery, particularly hepatobiliary and pancreatic surgery, has a high incidence of surgical site infection [6, 7]. It is reported that the incidence of surgical site infection in overall surgery is approximately 2–5% [6, 8]; in contrast, the incidence of surgical site infection in pancreaticoduodenectomy is as high as 10–30% [7, 9, 10]. Mortality rate for pancreaticoduodenectomy is estimated to be about 3% [11]. However, if surgical site infection occurs, mortality increases to 6%, and the hospital stay is extended by approximately 10 days [3]. Therefore, the prevention of surgical site infection in pancreaticoduodenectomy plays an important role in perioperative management.

Pancreaticoduodenectomy is the standard procedure for pancreatic or bile duct cancer in Japan. It is a technically complicated operation, involving delicate anastomosis for the reconstruction of the pancreatic duct and biliary tract [12]. Therefore, the surgical duration is long, and the procedure is highly invasive. Numerous studies have investigated the risk factors of surgical site infection in pancreaticoduodenectomy, and multiple risk factors (e.g., age, obesity, long surgical time, smoking, poor glycemic control, neoadjuvant chemotherapy, transfusion, biliary stent, and malnutrition) have been identified [3, 7, 9, 12–21].

However, whether anesthetic management is a risk factor is unclear, since only a few studies have assessed comprehensive risk factors, including anesthetic factors, in addition to preoperative patient factors and surgical factors in pancreaticoduodenectomy. It is considered that perioperative physiological changes in patients due to stress, fluid shift, and blood loss are more remarkable in pancreaticoduodenectomy compared to other, shorter abdominal surgeries.

The aim of this study was to investigate the perioperative and anesthetic risk factors of surgical site infection in pancreaticoduodenectomy.

## Materials and methods

### Design, setting, and patients

This was a single-center retrospective cohort study conducted at Yokohama City University Hospital, a 674-bed tertiary care center in Japan. Consecutive adult patients who underwent an elective open pancreaticoduodenectomy (including cases of resection of the portal vein or hepatic artery) between January 1, 2009 and March 31, 2018 were included. Patients who underwent additional resection of other organs were excluded. This study was approved by the institutional review board of Yokohama City University Hospital, and the study information was presented by opt-out (B190200010).

### Outcome

Primary outcome was the incidence of surgical site infection. The incidence of surgical site infection was determined from electronic medical records written by surgeons. Surgical site infection was classified based on the Japan Clinical Oncology Group postoperative complications criteria [22], which are based on the Clavien-Dindo classification [23]. Surgical site infection of Japan Clinical Oncology Group postoperative complications criteria grade 2 or higher was defined as the primary outcome, which was characterized by infection of the incision or intra-abdominal space requiring pharmacological or surgical treatments [22]. We also investigated the incidence of postoperative pancreatic fistula, a postoperative complication associated with space surgical site infections in pancreatic surgery [13, 24]. A postoperative pancreatic fistula was defined according to the International Study Group on Pancreatic Fistula guidelines [25]. We assessed the length of hospital stay of each patient and the number of in-hospital deaths.

### Candidate risk factors

The following candidate risk factors were collected from anesthetic charts and electronic medical records:

- Preoperative factors: age, sex, body mass index, year of surgery (before or after 2012; use of desflurane began in 2012 in our hospital), American Society of Anesthesiologists (ASA) classification, medical condition (cerebrovascular disease, chronic obstructive pulmonary disease, asthma, ischemic heart disease, heart failure, valvular disease, peripheral vascular disease, diabetes, liver cirrhosis, hypertension, alcoholism, neoadjuvant chemotherapy, biliary stent), medication (immunosuppressive drugs, statins), smoking status, and blood test data (albumin, C-reactive protein, total bilirubin, hemoglobin A1c [HbA1c])
- Intraoperative factors: type of surgery (reconstruction of the portal vein or hepatic artery), anesthetic agents (sevoflurane, desflurane, isoflurane, propofol), epidural anesthesia, use of vasopressors (norepinephrine, dopamine, dobutamine) and steroids, fraction of inspired O_2_, maximum serum glucose level, average temperature, duration of surgery, the volume of administered fluid (crystalloid, colloid, transfusion), blood loss, urine output, the dose of the local anesthetic agent (in epidural), the average mean blood pressure, and minimum hemoglobin level.

### Statistical analysis

The association between the postoperative outcome and each candidate risk factor was evaluated by univariate analysis, using the Mann-Whitney U test for continuous variables and Fisher’s exact test or the χ^2^ test for categorical variables.

A multivariable logistic regression analysis was performed with backward-stepwise variable selection to identify the risk factors of surgical site infection. A P value of <0.05 was considered statistically significant. The variables included in the multivariable analysis were those reported as risk factors in previous studies and those significantly correlated with the outcome in the univariate analysis. We confirmed the multicollinearity of the variables included in the multivariable analysis by variance inflation factors. All statistical analyses were performed using SPSS version 22 (IBM Corporation, Armonk, NY, USA).

## Results

**Fig 1:**
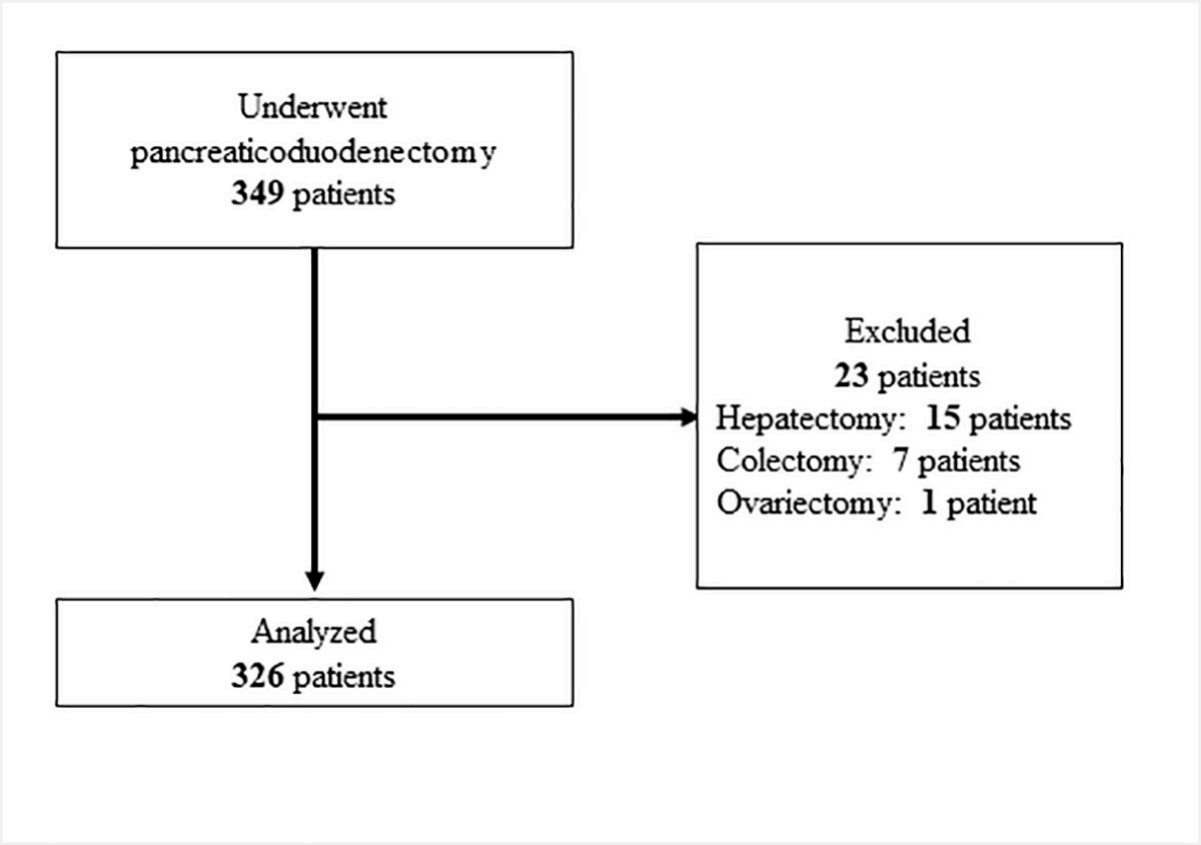
Flow chart of study enrollment.

A total of 349 patients underwent pancreaticoduodenectomy during the study period. Twenty-three patients were excluded as they required additional resection of other organs; hepatectomy (15 patients), colectomy (seven patients), and ovariectomy (one patient). Thus, 326 patients were included in the final analysis (Fig 1). The types of primary disease are presented in Table 1. Of the patients included in this study, 116 (35.6%) were women. The median age was 70 years (interquartile range; 64–75). The median duration of surgery was 10.9 hours (interquartile range; 9.5–12.4). The incidence of surgical site infection was 18.4% (60 patients). The incidence of each type and grade is presented in Table 2. The incidences of incisional and space surgical site infections were 15 and 48 cases, respectively. Three patients had both incisional and space surgical site infections.

**Table 1:** Types of primary disease.

Classification	n
**Pancreatic carcinoma**	170
**Bile duct carcinoma**	65
**Duodenal papillary carcinoma**	36
**Intraductal papillary-mucinous neoplasm**	31
**Pancreatic neuroendocrine tumor**	8
**Metastatic pancreatic tumor**	3
**Solid-pseudopapillary neoplasm**	3
**Serous cystic neoplasm**	3
**Mucinous cystic neoplasm**	2
**Gastrointestinal stromal tumor**	2
**Chronic pancreatitis**	2
**Arteriovenous aneurysm**	1

**Table 2:** Surgical site infection type and grade.

	Type	
JCOG PC criteria grade	Incisional SSI	Space SSI
**Ⅱ, n (%)**	14 (23.3%)	22 (36.7%)
**Ⅲa, n (%)**	1 (1.7%)	25 (41.7%)
**Ⅲb, n (%)**	0 (0.0%)	0 (0.0%)
**Ⅳa, n (%)**	0 (0.0%)	0 (0.0%)
**Ⅳb, n (%)**	0 (0.0%)	1 (1.7%)
**V, n (%)**	0 (0.0%)	0 (0.0%)

Three patients had both incisional SSI and space SSI.

JCOG PC criteria, Japan Clinical Oncology Group postoperative complications criteria; SSI, surgical site infection.

A total of 54 cases developed postoperative pancreatic fistula (Table 3). The incidence of postoperative pancreatic fistula was significantly higher in the group with surgical site infections than in the group without surgical site infections (22/60 [36.7%] vs 32/266 [12.0%]; odds ratio, 4.234; 95% confidence interval [CI], 2.228–8.045; P<0.001). In 272 cases without postoperative pancreatic fistula, 31 cases (11.4%) had space surgical site infections; in 54 cases with postoperative pancreatic fistula, 17 (31.5%) cases had space surgical site infections.

**Table 3:** Incidence of postoperative pancreatic fistula.

	SSI (-) (n = 266)		SSI (+)	
		Total	Space SSI (-)	Space SSI (+)
		(n = 60)	(n = 12)	(n = 48)
**POPF (all), n (%)**	32 (12.0)	22 (36.7)	5 (41.7)	17 (35.4)
**ISGPS Grade A, n**	2	0	0	0
**ISGPS Grade B, n**	26	17	5	12
**ISGPS Grade C, n**	4	5	0	5

"Space SSI (-)" means incisional SSI only without space SSI.

ISGPS, International Study Group on Pancreatic Fistula definition; SSI, surgical site infection; POPF, postoperative pancreatic fistula

The patients received intravenous prophylactic antibiotics within 60 min before the start of the operation. Flomoxef was administered as a prophylactic antibiotic every 3 hours during surgery. In patients with reduced renal function, the dosing interval was extended based on each patient’s estimated creatinine clearance [26]. According to the discretion of the attending surgeon, some patients were administered other types of prophylactic antibiotics (S1 Table). The data of the bacterial cultures obtained from the preoperative drained bile and the foci of infections are presented in S2 Table. Bacteria from the Enterobacteriaceae family such as *Enterococcus*, *Enterobacter*, and *Klebsiella* were found in the preoperative drained bile culture. In the cultures from the foci of infections, in addition to bacteria from the Enterobacteriaceae family, drug-resistant bacteria such as methicillin-resistant bacteria, *Pseudomonas*, *Stenotrophomonas*, and *Serratia* were observed. The antibiotics used for treatment of the surgical site infections are presented in S3 Table.

The median length of hospital stay was 48 and 26.5 days in the groups with and without surgical site infection, respectively; it was significantly prolonged in the group with surgical site infection compared to the group without (P<0.001) (Table 4). In-hospital deaths occurred in two patients, both of whom had surgical site infection.

**Table 4:** In-hospital death and length of hospital stay.

	Total	SSI (-) (n = 266)	SSI (+) (n = 60)
**In-hospital death, n (%)**	2 (0.6)	0 (0.0)	2 (3.3)
**Length of hospital stay (IQR, days)**	29 (22.0–43.8)	26.5 (21.0–38.0)	48 (33.8–65.5)

SSI, surgical site infection; IQR, interquartile range

### Preoperative characteristics

Preoperative characteristics are presented in Table 5. There were significant differences between the groups with and without surgical site infection regarding the year of surgery (odds ratio, 0.408; 95% CI, 0.226–0.734; P = 0.002), cerebrovascular disease (odds ratio, 3.434; 95% CI, 1.394–8.461; P = 0.009), ischemic heart disease (odds ratio, 12.000; 95% CI, 2.269–63.451; P = 0.003), hypertension (odds ratio, 1.800; 95% CI, 1.023–3.166; P = 0.04), and serum albumin level (odds ratio, 1.962; 95% CI, 1.086–3.545; P = 0.024).

**Table 5:** Preoperative characteristics.

Categorical variables	SSI (-) (n = 266)	SSI (+) (n = 60)	Odds ratio	95% CI	P value
**Age ≥70 years, n (%)**	129 (48.5)	35 (58.3)	1.487	0.844–2.621	0.169
**Female, n (%)**	91 (34.2)	25 (41.7)	1.374	0.775–2.435	0.276
**BMI ≥25, n (%)**	34 (12.8)	10 (16.7)	1.365	0.633–2.943	0.426
**Year of surgery after 2012, n (%)**	206 (77.4)	35 (58.3)	0.408	0.226–0.734	0.002
**ASA classification, n (%)**					
** 1,2**	253 (95.1)	57 (95.0)	ref.	―	―
** ≥3**	13 (4.9)	3 (5.0)	1.024	0.283–3.713	0.591
**Medical condition, n (%)**					
** Cerebrovascular disease**	13 (4.9)	9 (15.0)	3.434	1.394–8.461	0.009
** Chronic obstructive pulmonary disease, asthma**	17 (6.4)	2 (3.3)	0.505	0.114–2.247	0.285
** Ischemic heart disease**	2 (0.8)	5 (8.3)	12.000	2.269–63.451	0.003
** Heart failure**	2 (0.8)	0 (0.0)	NA		
** Valvular disease**	14 (5.3)	2 (3.3)	0.621	0.137–2.806	0.409
** Peripheral vascular disease**	3 (1.1)	0 (0.0)	NA		
** Diabetes**	98 (36.8)	21 (35.0)	0.923	0.514–1.659	0.789
** Liver cirrhosis**	3 (1.1)	0 (0.0)	NA		
** Hypertension**	95 (35.7)	30 (50.0)	1.800	1.023–3.166	0.040
** Alcoholism**	2 (0.8)	0 (0.0)	NA		
** Neoadjuvant chemotherapy**	108 (40.6)	18 (30.0)	0.627	0.343–1.147	0.128
** Biliary stent**	104 (39.1)	24 (40.0)	1.038	0.586–1.840	0.897
**Medication, n (%)**					
** Immunosuppressive drug**	5 (1.9)	2 (3.3)	1.800	0.341–9.508	0.379
** Statin**	53 (19.9)	15 (25.0)	1.340	0.694–2.585	0.382
**Smoking status, n (%)**					
** Never smoker**	92 (34.6)	19 (31.7)	ref.	―	―
** Former smoker**	134 (50.4)	31 (51.7)	1.120	0.597–2.103	0.724
** Current smoker**	24 (9.0)	5 (8.3)	1.009	0.342–2.979	0.589
** Unknown**	16 (6.0)	5 (8.3)	1.513	0.494–4.633	0.324
**Examination, n (%)**					
** Albumin <3.5 g/dL**	64 (24.1)	23 (38.3)	1.962	1.086–3.545	0.024
** C-reactive protein >1.0 mg/dL**	37 (13.9)	14 (23.3)	1.884	0.943–3.762	0.070
** T-Bil >1.0 mg/dL**	28 (10.5)	10 (16.7)	1.700	0.776–3.723	0.181
** HbA1c >7.0%**	38 (14.3)	5 (8.3)	0.574	0.215–1.531	0.262

SSI, surgical site infection; CI, confidence interval; BMI, body mass index; ASA, American Society of Anesthesiologists; ref, reference; NA, Not applicable; T-Bil, total bilirubin; HbA1c, hemoglobin A1c.

### Intraoperative information

Intraoperative information is presented in Table 6. In this cohort, anesthetic management was entrusted by each attending anesthesiologist. In most cases, volatile anesthetics were used to maintain anesthesia, and epidural anesthesia was used for postoperative analgesia. There were no significant differences between the groups with and without surgical site infection regarding the duration of surgery, intraoperative fluid and transfusion volumes, blood loss, body temperature, minimum hemoglobin level, glycemic control, the average mean blood pressure, and the use of any vasopressor or steroid (used for prevention of postoperative nausea and vomiting or reconstruction of the portal vein or hepatic artery). Only the use of desflurane significantly differed between the two groups (odds ratio, 0.369; 95% CI, 0.198–0.689; P = 0.001).

**Table 6:** Intraoperative information.

Categorical variables	SSI (-) (n = 266)	SSI (+) (n = 60)	Odds ratio	95% CI	P value
**Type of surgery, n (%)**					
** Reconstruction of the portal vein**	124 (46.6)	20 (33.3)	0.573	0.318–1.031	0.061
** Reconstruction of the hepatic artery**	32 (12.0)	7 (11.7)	0.966	0.404–2.306	0.938
**Anesthetic agents, n (%)**					
** Sevoflurane**	128 (48.1)	43 (71.7)	ref.	―	―
** Desflurane**	129 (48.5)	16 (26.7)	0.369	0.198–0.689	0.001
** Isoflurane**	2 (0.8)	0 (0.0)	NA		
** Propofol**	7 (2.6)	1 (1.7)	0.425	0.051–3.556	0.372
**Epidural anesthesia, n (%)**	255 (95.9)	58 (96.7)	1.251	0.270–5.797	0.560
**Medication, n (%)**					
** Norepinephrine**	101 (38.0)	20 (33.3)	0.817	0.452–1.475	0.502
** Dopamine**	13 (4.9)	2 (3.3)	0.671	0.147–3.055	0.456
** Dobutamine**	20 (7.5)	4 (6.7)	0.879	0.289–2.671	0.538
** Steroid**	117 (44.0)	26 (43.3)	0.974	0.553–1.714	0.927
**FiO2 ≤0.4, n (%)**	49 (18.4)	15 (25.0)	1.476	0.762–2.860	0.247
**Maximum serum glucose >180 mg/dL, n (%)**	127 (47.7)	31 (51.7)	1.170	0.668–2.049	0.583
**Average of temperature <36.0°C, n (%)**	84 (31.6)	17 (28.3)	0.857	0.462–1.589	0.623
**Transfusion, n (%)**	77 (28.9)	16 (26.7)	0.893	0.475–1.677	0.724
**Continuous variables**	**SSI (-) (n = 266)**		**SSI (+) (n = 60)**		
	**Median**	**IQR**	**Median**	**IQR**	**P value**
**Duration of surgery (h)**	10.9	9.5–12.4	11.1	9.8–12.5	0.308
**Volume of administered fluid (ml)**					
** Crystalloid**	5575	4400–6850	5925	4988–7300	0.076
** Colloid (including 5% albumin)**	1000	500–1500	1000	500–1250	0.270
** Transfusion**	0	0–280	0	0–280	0.715
** Total (crystalloid, colloid, and transfusion)**	6850	5551–8400	6975	5940–8788	0.365
** Fluid balance**	4556	3594–5622	4598	3951–5478	0.798
**Infusion rate (ml/kg/h)**					
** Crystalloid**	9.4	7.5–11.4	9.5	8.0–11.3	0.539
** Total (crystalloid, colloid, and transfusion)**	11.5	9.5–14.2	11.4	9.2–13.4	0.711
** Fluid balance**	7.6	6.1–9.2	7.4	6.1–9.0	0.502
**Blood loss (ml)**	803	465–1277	754	449–1445	0.549
**Urine output (ml/kg/h)**	1.9	1.3–2.9	2.2	1.3–3.1	0.450
Local anesthetic agents (in epidural) (ml/h)*	51.8	29.0–72.4	49.7	24.4–69.2	0.309
**Average of mean blood pressure (mmHg)**	69	65–73	68	64–72	0.434
**Minimum hemoglobin (g/dL)**	9.3	8.3–10.4	9.5	8.2–10.8	0.483

SSI, surgical site infection; CI, confidence interval; FiO2, fraction of inspired O2; ref, reference; NA, Not applicable.

*The dose of local anesthetic agents administered in epidural was converted to that of lidocaine (10 mg lidocaine = 10 mg mepivacaine = 2.5 mg bupivacaine = 2 mg ropivacaine = 1.25 mg levobupivacaine). SSI, surgical site infection; IQR, interquartile range.

### Multivariable analysis

The following variables were included in the multivariable logistic regression analysis:

- Risk factors reported in previous studies: body mass index ≧25 kg/m^2^ [9, 13, 18], ASA classification grade ≧3 [12], duration of surgery [7, 9, 12, 13, 18, 19], age ≧70 years [7, 9, 18], transfusion [9, 17, 18], neoadjuvant chemotherapy [16], preoperative biliary stent [12, 16], smoking [18], preoperative and intraoperative poor glycemic control (preoperative HbA1c level ≧7.0%, intraoperative maximum serum glucose level ≧180 mg/dL) [18], and serum albumin level <3.5 g/dL [18].
- Factors that correlated with outcomes at the level of P<0.05 in univariate analysis: year of surgery after 2012, cardiovascular disease, ischemic heart disease, hypertension, and use of desflurane (reference is the use of sevoflurane).

The multivariable analysis revealed that desflurane was associated with less surgical site infections than sevoflurane (odds ratio, 0.503; 95% CI, 0.260–0.973; P = 0.041). In contrast, the duration of surgery (odds ratio, 1.162; 95% CI, 1.017–1.328; P = 0.027) and the presence of cerebrovascular disease (odds ratio, 3.544; 95% CI, 1.326–9.469; P = 0.012) and ischemic heart disease (odds ratio, 10.839; 95% CI, 1.887–62.294; P = 0.008) were identified as risk factors of surgical site infection (Table 7).

**Table 7:** Multivariable logistic regression analysis.

Variables	Odds ratio	95% Confidence interval			P value
**Desflurane use**	0.503	0.260	-	0.973	0.041
**Duration of surgery (h)**	1.162	1.017	-	1.328	0.027
**Cerebrovascular disease**	3.544	1.326	-	9.469	0.012
**Ischemic heart disease**	10.839	1.887	-	62.249	0.008

## Discussion

We investigated the incidence of surgical site infection and the associated risk factors, including anesthetic factors, in pancreaticoduodenectomy, a prolonged abdominal surgery. The incidence of surgical site infection in pancreaticoduodenectomy is high and associated with increased rates of reoperation, prolonged hospital stay, and increased in-hospital death, and affects patient prognosis [3]. The incidence of surgical site infection in this study was 18.4%, which is similar to that reported in previous studies [7, 9, 10]. In this study, the length of hospital stay was prolonged in the group with surgical site infection, and both patients who died postoperatively developed surgical site infections. This study yielded two main conclusions: first, the use of desflurane was a possible protective factor against surgical site infection. Second, the duration of surgery and presence of cerebrovascular disease and ischemic heart disease were possible risk factors for surgical site infection.

Previous studies examining the association between surgical site infection and volatile anesthetics have been limited. Volatile anesthetics include isoflurane, sevoflurane, desflurane, etc. Currently, sevoflurane and desflurane are mainly used in Japan. Koutsogiannaki et al. [27] examined the effects of isoflurane and propofol exposure on surgical site infection by creating a mouse surgical site infection model infected with *Staphylococcus aureus*. They demonstrated that mice exposed to isoflurane for six hours had more bacterial growth than mice that did not receive isoflurane or mice that were exposed to isoflurane for only two hours. Koo et al. [28] retrospectively investigated the incidence of surgical site infection after colorectal surgery and found that patients anesthetized with volatile anesthetics (sevoflurane or desflurane) had a higher incidence of surgical site infection than that in patients anesthetized with propofol. Shimizu et al. [29] retrospectively compared the incidence of surgical site infection after elective open gastrointestinal surgery in two propensity-matched groups anesthetized with sevoflurane or propofol. In contrast to the above results, they demonstrated that the incidence of surgical site infection after sevoflurane anesthesia was lower than that after propofol anesthesia. Several mechanisms have been proposed to explain the association between anesthetics and surgical site infection. Neutrophils play many roles in host defense [30], and it is speculated that anesthetics modify these important roles, such as phagocytosis or production of oxygen radicals [27–29]. Some studies have observed neutrophil dysfunction after exposure to volatile anesthetics, but the clinical effects in humans are not clear [30]. The present study has demonstrated the differential effects of desflurane and sevoflurane on the incidence of surgical site infection. To the best of our knowledge, no previous studies have compared desflurane with other anesthetics to examine the effect on surgical site infection. Further basic and clinical research is needed.

In this study, sevoflurane was used throughout the study period, but desflurane was used only after 2012 when it was introduced to our hospital (desflurane was introduced to the Japanese market in 2011). This is in marked contrast to the U.S. where sevoflurane was introduced into clinical practice later than desflurane. Therefore, the beneficial effects of desflurane compared to sevoflurane demonstrated by our study may be explained by differences in the period of surgery (i.e., surgeries performed more recently were associated with a low incidence of surgical site infection because of technical and/or medical advances, but also because the patients were more likely to be anesthetized with desflurane). Thus, the year of surgery (before or after starting to use desflurane in our hospital) was included in the multivariable logistic regression analysis as a confounding factor. Nevertheless, desflurane was still associated with fewer surgical site infections than sevoflurane.

In this study, ischemic heart disease and cerebrovascular diseases were identified as risk factors. Kent et al. [31] prospectively investigated the incidence of infectious complications after elective pancreatectomy and its predictors in 550 cases. They reported that coronary artery disease was a predictor of surgical site infection. It is suggested that the increase in oxygen supply to surgical sites may prevent surgical site infection. Reducing the partial pressure of oxygen at the wound site reduces the neutrophils’ ability to kill bacteria [32]. Oxidative killing plays an important role in protecting the wound site from bacterial infection; therefore, the tissue partial pressure of oxygen and the risk of infection are inversely correlated [33]. In patients with these risk factors, the oxygen supply to surgical sites may be reduced because of microcirculatory failure due to arteriosclerosis.

This study has several limitations. First, patient selection may have been biased toward difficult or complicated cases of pancreaticoduodenectomy because this study was performed at a single tertiary care center. The generalizability of the findings is also limited. Second, we defined the outcome based on the Japan Clinical Oncology Group postoperative complications criteria, but could not confirm that the diagnosis of surgical site infection followed the Centers for Disease Control and Prevention/ National Healthcare Safety Network surveillance definition [34]. Third, we included neoadjuvant chemotherapy and biliary stenting in the multivariable logistic analysis but did not include differences in the staging, because the staging could not be evaluated with the same index due to differences in the primary disease and the year of surgery. Therefore, the effects of these factors were not considered.

## Conclusions

We found that desflurane was better than sevoflurane in preventing surgical site infection in pancreaticoduodenectomy and that the duration of surgery and presence of cerebrovascular disease and ischemic heart disease were newly identified possible new risk factors of surgical site infection in pancreaticoduodenectomy. However, the mechanism underlying the effect of these factors on surgical site infection is unresolved. Further research is needed to confirm the association between these factors and surgical site infection.

## Supporting information

S1 Table Types of prophylactic antibiotics. (DOCX)

S2 Table Bacterial culture data. (DOCX)

S3 Table Types of antibiotics for treatment of surgical site infections. (DOCX)
