# Supplementary material for: Perioperative and anesthetic risk factors of surgical site infection in patients undergoing pancreaticoduodenectomy: A retrospective cohort study
Source: PLoS One. 2020 Oct 14;15(10):e0240490. doi: 10.1371/journal.pone.0240490 (PMC7556444; doi:10.1371/journal.pone.0240490)
Supplement: S1 Table — (DOCX) [file pone.0240490.s001.docx]

**Supplementary Table 1. Types of prophylactic antibiotics.**

| **Antibiotics** | **n** |
| --- | --- |
| **Flomoxef** | 300 |
| **Piperacillin/tazobactam** | 13 |
| **Cefoperazone/sulbactam** | 2 |
| **Doripenem** | 2 |
| **Cefazolin** | 2 |
| **Ciprofloxacin** | 2 |
| **Ampicillin** | 1 |
| **Ampicillin/sulbactam** | 1 |
| **Vancomycin** | 1 |
| **Clindamycin** | 1 |
| **Minocycline** | 1 |
