# Supplementary material for: Perioperative and anesthetic risk factors of surgical site infection in patients undergoing pancreaticoduodenectomy: A retrospective cohort study
Source: PLoS One. 2020 Oct 14;15(10):e0240490. doi: 10.1371/journal.pone.0240490 (PMC7556444; doi:10.1371/journal.pone.0240490)
Supplement: S2 Table — (DOCX) [file pone.0240490.s002.docx]

**Supplementary Table 2. Bacterial culture data.**

|  | **Drained bile culture (n=12)** | **Wound culture (n=12)** | **Intraabdominal culture  (n=60)** |
| --- | --- | --- | --- |
| ***Enterococcus faecalis*** | 3 | 0 | 22 |
| ***Enterococcus faecium*** | 3 | 0 | 20 |
| ***Enterobacter cloacae*** | 5 | 2 | 16 |
| ***Candida albicans*** | 0 | 0 | 11 |
| ***Pseudomonas aeruginosa*** | 1 | 1 | 10 |
| ***Stenotrophomonas maltophilia*** | 0 | 0 | 10 |
| ***Staphylococcus aureus***  **(methicillin-resistant)** | 0 | 1 | 8 |
| ***Klebsiella pneumoniae*** | 6 | 0 | 7 |
| ***Corynebacterium sp.*** | 0 | 1 | 6 |
| ***Escherichia coli*** | 2 | 0 | 6 |
| ***Enterobacter aerogenes*** | 0 | 0 | 5 |
| ***Staphylococcus epidermidis***  **(methicillin-resistant)** | 0 | 0 | 5 |
| ***Candida glabrata*** | 0 | 0 | 4 |
| ***Serratia marcescens*** | 0 | 0 | 4 |
| ***Staphylococcus haemolyticus***  **(methicillin-resistant)** | 0 | 0 | 4 |
| **Others** | 1 | 1 | 21 |

The numbers include overlap in patients.
