# Supplementary material for: Perioperative and anesthetic risk factors of surgical site infection in patients undergoing pancreaticoduodenectomy: A retrospective cohort study
Source: PLoS One. 2020 Oct 14;15(10):e0240490. doi: 10.1371/journal.pone.0240490 (PMC7556444; doi:10.1371/journal.pone.0240490)
Supplement: S3 Table — (DOCX) [file pone.0240490.s003.docx]

**Supplementary Table 3. Types of antibiotics for treatment of surgical site infections.**

| **Antibiotics** | **n** |
| --- | --- |
| **Piperacillin/tazobactam** | 34 |
| **Meropenem** | 23 |
| **Doripenem** | 16 |
| **Imipenem/cilastatin** | 8 |
| **Biapenem** | 1 |
| **Cefepime** | 4 |
| **Vancomycin** | 37 |
| **Linezolid** | 5 |
| **Daptomycin** | 1 |
| **Ciprofloxacin** | 1 |
| **Levofloxacin** | 3 |
| **Gentamicin** | 1 |
| **Clindamycin** | 3 |
| **Micafungin** | 12 |
| **Fluconazole** | 4 |
| **Others** | 5 |

The numbers include overlap in patients.
